# Experience of Snakebite Envenomation by a Desert Viper in Qatar

**DOI:** 10.1155/2020/8810741

**Published:** 2020-10-12

**Authors:** Amr Elmoheen, Waleed Awad Salem, Mahmoud Haddad, Khalid Bashir, Stephen H Thomas

**Affiliations:** ^1^Department of Emergency Medicine, Hamad Medical Corporation, Doha, Qatar; ^2^Weill Cornell Medical College in Qatar, Doha, Qatar; ^3^Barts and The London School of Medicine, Queen Mary University of London, London, UK

## Abstract

Crotaline and elapid snakebites are reported all over the world as well as in the Middle East and other countries around this region. However, data regarding snakebites and their treatment in Qatar are limited. This review paper is going to investigate the presentation and treatment of snakebite in Qatar. A good assessment helps to decide on the management of the snakebites envenomation. Antivenom and conservative management are the mainstays of treatment for crotaline snakebite. Point-of-care ultrasound (POCUS) has been suggested to do early diagnosis and treatment of soft tissue problems, such as edema and compartment syndrome, after a snakebite. The supporting data are not sufficient regarding the efficiency of POCUS in diagnosing the extent and severity of tissue involvement and its ultimate effect on the outcome. Further research is suggested in this case. Systemic complications, such as bleeding diathesis, can be managed by administering clotting factors and platelets.

## 1. Introduction

Snakebites are experienced worldwide, but data regarding snakebites and their treatment in Qatar are limited. Crotaline and elapid snakebites are reported all over the world as well as in the Middle East and other countries around this region [1–4]. However, a few cases of snakebites are reported in Qatar per year, making them less prevalent [5]. Elapid envenomation is rare in Qatar, so that crotaline bites can be a concern. Crotaline snakebites lead to neurotoxicity, as reported in many studies [6–8]. However, no such incident has been seen in Qatar so far. That is why other symptoms of the crotaline bite are focused more.

**Figure 1:**
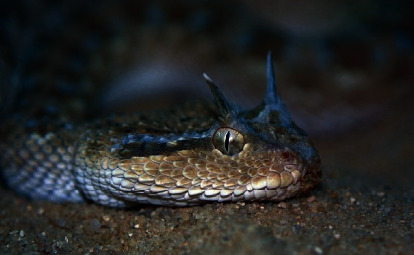
*Cerastes gasperettii* (horned) (licensed under the Creative Commons Attribution 3.0 Unported License).

In many countries, including Qatar, where elapid bites are almost unreported, the identification of the snake genus and species is less important than carrying out clinical assessment and diagnosis crotaline snakebite. A good assessment helps to decide about the administration of antivenom that is the first-line treatment for snakebite in Qatar. The Saharan horned viper called *Cerastes cerastes* [2] has about the same distribution in Qatar as *Cerastes gasperettii* [9] (Figure 1).

*Cerastes* snakes are called desert vipers, and they have similarities with related species in many ways, including having a similar emergency treatment, so differentiating between the species is not necessary. The horned vipers have an eponymous supraorbital horn that can also be absent in some cases. Large head, overall length up to 3 feet, dorsal markings with a white ventral surface, and sudden taper to a black tail are characters of a *Cerastes* species. *Cerastes* are the most common snakes in the deserts of North Africa and the Middle East [2]. Bites from *Cerastes* can be experienced from April to October while they hibernate from November through March [10, 11]. This review paper is going to investigate the various presentation, diagnosis, and treatment of snakebite in Qatar. Such a review is aimed to share the experience and facilitate the future management of snakebite cases.

## 2. Presentation of *Cerastes* Envenomation in Humans

In the case of *Cerastes* envenomation, a patient can have both local and systemic symptoms. Locally, it causes pain and swelling, while systemically, it causes hematoma formation, which further leads to many complications [2, 12]. The severity of snakebite envenomation is summarized in Table 1.

Investigations of this type of snake envenomation should include serial measurements of prothrombin time, hemoglobin, platelet, and fibrinogen, which is more sensitive and can help in early detection of hematologic venom effects [13]. As there are a significant number of cases of false-negative INR results in patients with severe venous-induced consumption coagulopathy, point-of-care testing for INR or d-Dimer should not be used in snakebite patients [14]. Studies conducted on the patients of snakebite by Saharan vipers show other consequences, including fibrinolysis, thrombocytopenia, microangiopathic hemolytic anemia, and acute renal failure. The venom has enzymes called serine proteases (e.g., cerastotin) and other proteins like cerastocytin that work to activate factor X, platelet aggregators, and nephrotoxins leading to TTP and HUS [2, 15–17]. As the systemic involvement increases with time, early intervention can save the patient's life. These snakebites are usually not life-threatening if preventive measures, such as binding the affected area with tourniquet and antivenom administration, are taken in time [2]. In the regional studies, the mortality rate reported varies from 0 to 3.7% death. The children's mortality within the subpopulation was almost double of the adults. No specification of the types of snakes was found, and the health workers could not identify the types of the snake [3].

## 3. General Measures in Snakebites

Like in any other life-threatening emergency, the management of snakebite also starts with some general measures that should be done even before the patient is shifted to a medical facility. A patient airway should be ensured, and breathing and circulatory problems should be addressed. Prehospital interventions are of vital importance in areas where transport to a hospital takes a long time. However, that is not the case with Qatar as transport time is short, so the need for prehospital interventions is limited. The crotaline snakes found in Qatar have not been reported to cause neurotoxicity, so securing the airway and supporting breathing is not required. However, circulatory support might be needed in some patients, and it follows the same principles as elsewhere. No vigorous fluid administration should be done unless the patient has volume depletion or hemorrhagic complications. Otherwise, it can lead to clotting factor dilution and further hemorrhage.

Applying a tourniquet can hinder the blood supply and worsen the symptoms like edema. The blockage of venous blood drainage can lead to deep venous thrombosis (DVT), pulmonary embolism, and stroke [15]. Moreover, studies also report the bad outcome of early interventions like incising wounds and extracting venom [15].

Importantly, there is a need to monitor neurological symptoms in a patient of snakebite because there is a risk of hemorrhagic and ischemic stroke that can cause drowsiness and other symptoms. Crotaline-induced hematotoxicity can cause the risk of stroke [18].

### 3.1. Use of Antivenom to Treat Snakebite

Crotaline snakebites can be treated with antivenoms. Crotaline bites in Qatar are treated using an antivenom, which is effective against six different snakebites. The antivenom is produced by hyperimmunizing Arabian horses [19].

Antivenom administration is not required in every patient, so deciding if it is needed in a patient or not is important. The decision is made based on clinical findings. Some rules are followed while making a judgment about antivenom use. In crotaline snakebite, antivenom is given if swelling and pain extend beyond the site of the bite, there is coagulation profile derangement, and there is hemorrhage or hemodynamic instability [15].

Antivenom administration is influenced by various situational factors. In some cases, antivenom is given even in the presence of only one of the abovementioned factors. For example, the administration of antivenom has been reported in Oman in the presence of progressive swelling after carpet viper bite [1].

In case of a life-threatening emergency caused by snakebite, there are no absolute contraindications to antivenom administration. However, the patient's atopy should be kept in mind. Also, if there is a history of an allergic reaction to a similar product previously, then precautions should be taken. Such patients can be given corticosteroids, antihistaminics, or epinephrine before giving antivenom to avoid any kind of allergic reaction [15].

The antivenom used in Qatar is a highly purified preparation containing F(ab') 2 fractions of immunoglobulins raised against venoms of six snakes (including pit vipers and elapids). The antivenom is obtained via hyperimmunization of Arabian horses to venoms from *Bitis arietans*, *Echis coloratus*, *Echis carinatus*, *Naija haje*, *Walterinnesia aegyptia*, and *Cerastes cerastes* [20].

### 3.2. Antivenom Administration

In Qatar, the same protocol is followed for antivenom administration as in other countries. Four to six vials of polyvalent antivenom are diluted in saline fluid and are given intravenously over an hour. Also, the patient needs to be kept under observation during and after the administration. An anaphylactic reaction can be a complication of antivenom administration that should be looked out for and should be managed immediately. Patients who are at high risk of any sort of allergic reaction should be treated with antihistaminics, epinephrine, or corticosteroids before the initiation of antivenom infusion. Administering test doses does not help in the case of antivenoms because they do not help identify the patients who can have an allergy [21]. Studies have shown that pretreatment with diluted antivenom has no value in predicting early or late allergic reactions [15]. However, after an allergic reaction, the patient can be immediately treated with antihistaminics, corticosteroids, or epinephrine, and the infusion can be restarted at a slower rate [22, 23].

The decision of antivenom dose should be repeated or not is made according to the response to initial therapy. This response differs according to the snakebite and antivenom administered. In crotaline snakebite, coagulopathy is reversed within six hours of antivenom injection. A second dose of antivenom can be given if there are persistent systemic symptoms even after one hour of antivenom dose or if the coagulopathy is not corrected within six hours [15]. Just as the decision about antivenom injection is subjective, the second dose in a patient with improving symptoms or an allergic reaction is usually avoided, and conservative therapy is done instead.

### 3.3. Allergic Reactions to Crotaline Antivenom

With older antivenom preparations, allergic reactions were quite common that had put a question mark on the usage of antivenoms [24]. Different prevalence and rate of allergic reactions have been reported in different studies. The adverse reactions can range from less than 5% to 20% or higher. However, the reports of allergic reactions antivenom are at the lower end of this range while using the recent preparations of antivenoms [21, 25, 26]. Crotaline antivenom has also reported less adverse reactions, and it can be attributed to the recent understanding of the importance of a slow infusion rate for the prevention of allergic reactions [22].

The allergic reaction can be early or delayed. The early ones appear after 10 to 180 minutes of giving antivenom. The symptoms include itching, hive formation, tachycardia, nausea, and vomiting. This should be treated like an anaphylactic reaction with epinephrine, antihistaminics, and corticosteroids [15, 22, 24]. However, when the patient only has a fever, it can be a pyrogenic reaction due to contamination of antivenom with some endotoxin-like chemical. Antivenom infusion should not be stopped, and fever should be treated with paracetamol [15].

Some people can develop a delayed allergic reaction that is called serum sickness. It is due to IgG-mediated immune response to proteins present in exogenous serums. The symptoms can differ depending on the severity of the reaction. A trial was conducted in 2016 in Australia, with over 100 cases diagnosed with serum sickness using criteria; the presence of at least three of the following after 5–20 days of antivenom administration can be labeled as serum sickness: (1) fever, (2) erythematous rash or urticaria, (3) arthralgia or myalgia, (4) headache, (5) malaise, and (6) nausea or vomiting [27].

As serum sickness is a delayed reaction, patients should be educated about its symptoms upon discharge. Moreover, the patient is asked about symptoms during follow-ups. The reaction is not severe and can be managed without hospital stay with the help of antihistaminics and corticosteroids [15, 28].

### 3.4. Complications after Crotaline Snakebite

There are two major types of complications that are faced after crotaline envenomation: local and systemic.

In local complications, wound infection and compartment syndrome are very important. Normal wound care is done, and there is no special instruction regarding the snakebite wound. Also, a tetanus injection should be given. These steps are taken to avoid wound infection. The affected limb is slightly elevated to prevent blood pooling and swelling. Too much elevation is not carried out to avoid discomfort and compromised blood flow. This elevation is useless in case of obstruction down the draining path [15]. Also, the patient should be evaluated for venous thrombosis in the affected limb due to slow blood flow. It can happen even when the patient is experiencing hemorrhage in the rest of the body [29]. Antibiotics are commonly used in snakebite cases to prevent wound infection. According to research, half of the snakebite cases are given antibiotics in the USA [30]. However, the latest research shows that there is no role of antibiotic prophylaxis in snakebite cases [15].

The number of clinical trials to determine the role of antibiotic prophylaxis is very less. Therefore, the role of the short-term antibiotic course to prevent wound infection cannot be ruled out. It is especially true for the punctured wound caused by the fangs of a snake. Such wounds cannot be cleaned easily and have a higher tendency of infection. Antibiotics can be helpful in such cases [31].

The most important systemic complication of crotaline snakebite is bleeding complications. Hematoxins are present in crotaline snakes, and they can vary. Still, *Cerastes* causes rapid coagulopathy and microangiopathic hemolytic anemia that can be evident in complete blood count (including platelet count), prothrombin time, and fibrinogen levels clinically [2]. Hematotoxicity takes just a few hours to develop [15].

No prophylactic treatment, such as heparin or antifibrinolytics, is recommended for this complication [15]. Once the patient develops bleeding complications, clotting factors and platelets are replaced as required. As the patient may also develop deep venous thrombosis (DVT), early use of these clotting factors, etc., is avoided [29].

### 3.5. Point-of-Care Ultrasound (POCUS) in Snakebite

POCUS carries an important place in snakebite treatment as it helps diagnose edema, compartment syndrome, and cellulitis [31]. It has been suggested that POCUS can be helpful in early diagnosis of tissue involvement that is unnoticeable otherwise. However, this needs to be researched further. If further studies confirm the role of POCUS in identifying the levels of edema as a predictive of the severity of soft tissue involvement in crotaline envenomation, then POCUS could help prioritize snakebite patients and guide earlier administration of antivenom.

Previous studies have reported that POCUS can be potentially used as a helpful tool in snakebites that cause soft tissue destruction. In California, the ultrasound performed in the emergency department showed both superficial structural involvement and sparing of deeper muscle and tissues in patients with subcutaneous edema after the crotaline snakebite [32]. In South Africa, POCUS helped demonstrate the severity of edema in snake-bitten limbs as compared to the normal limb [33, 34]. However, none of these papers specifically investigated if POCUS is helpful as a predictor of the ultimate level of tissue involvement.

## 4. Conclusion

Snakebites are uncommon in Qatar. However, a few cases are reported every year regardless, and crotaline snakebites are common. A similar protocol is followed for the treatment of snakebite all over the world. As soon as a patient reports snakebite, he is taken under the care, and general measures are taken if required. Once the patient is shifted to a healthcare facility, antivenom is considered to prevent complications of snakebite. Antivenom and conservative management are the mainstays of treatment for crotaline snakebite. The dose of antivenom can be repeated based on the patient's clinical assessment. The decision is subjective. Also, it is crucial to keep an eye on early and delayed allergic reactions caused by antivenoms. Patients can still develop local and systemic complications of snakebite even after antivenom dose. It is vital to carry out general wound care to avoid wound infection. The role of prophylactic antibiotics is not well established and needs further research. POCUS has been suggested to do early diagnosis and treatment of soft tissue problems, such as edema and compartment syndrome, after a snakebite. Again, the supporting data are not sufficient regarding the efficiency of POCUS in diagnosing the extent and severity of tissue involvement and its ultimate effect on the outcome. Further research is suggested in this case. Systemic complications, such as bleeding diathesis, can be managed by administering clotting factors and platelets.

## Figures and Tables

**Table 1:** Assessment of severity of envenomation.

No envenomation	Absence of local or systemic reactions; fang marks (±)
Mild envenomation	Fang marks (+), moderate pain, minimal local edema, erythema (+), ecchymosis (±), and no systemic reactions (hemoglobin, platelets, prothrombin time, and fibrinogen are normal)
Moderate envenomation	Fang marks (+), severe pain, moderate local edema erythema and ecchymosis (+), systemic weakness, sweating, syncope, nausea, vomiting, anemia, or thrombocytopenia
Severe envenomation	Fang marks (+), severe pain, severe local edema, erythema and ecchymosis (+), hypotension, paresthesia, coma, pulmonary edema, and respiratory failure

## Data Availability

The authors ensure that data are available on request.
